# *Mycobacterium tuberculosis* as a cause of mandibular osteomyelitis in a young woman: a case report

**DOI:** 10.1186/s13256-016-1118-x

**Published:** 2016-12-20

**Authors:** Jorge Tellez-Rodriguez, Rubi Lopez-Fernandez, Rodolfo Rodriguez-Jurado, Hayde Nallely Moreno-Sandoval, Francisco Martinez-Perez, Juan Antonio Gonzalez-Barrios

**Affiliations:** 1 Departamento de Cirugía Maxilofacial, Insurgentes sur 3700, Letra C, Col. Insurgentes Cuicuilco, Mexico City, Mexico; 2 Departamento de Anatomía Patológica del Instituto Nacional de Pediatría, Insurgentes sur 3700, Letra C, Col. Insurgentes Cuicuilco, Mexico City, Mexico; 3 Laboratorio de Medicina Genómica, Hospital Regional “1o. de Octubre”, Avenida Instituto Politécnico Nacional 1669, Mexico City, 07760 Mexico; 4 Laboratorio de Genómica de Celomados, Grupo de Investiagición en Microbiología y Genética, Escuela de Biología, Universidad Industrial de Santander, Carrera 27, Calle 9, Bucaramanga, 680002 Santander Colombia

**Keywords:** Tuberculosis, Osteomyelitis, *Mycobacterium tuberculosis*, Jaw, Case report

## Abstract

**Background:**

Tuberculosis is considered an emerging disease worldwide; in the last 10 years, its incidence has increased to more than 9.6 million cases of active tuberculosis. In 2014, it resulted in 1.5 million patient deaths. However, oral presentation with bone involvement occurs in less than 3% of all reported cases and rarely arouses clinical suspicion on initial presentation.

**Case presentation:**

A 15-year-old Mexican girl who had a previous diagnosis of neurofibromatosis presented to our hospital with pain and swelling in the region of the left mandibular body since November 2011. A clinical examination revealed pain in the mandibular region, a mass of soft consistency that seemed to involve bone, and a fistula with discharge of intraoral purulent material. Additionally, tachycardia and hyperthermia were observed. The left submental and submandibular regions had a 12-cm-diameter swelling, which was well-delineated and nonerythematous. The final diagnosis was established by real-time polymerase chain reaction.

**Conclusions:**

The final diagnosis of rare cases of tuberculous osteomyelitis in the jaw can be established by deoxyribonucleic acid (DNA) identification of *Mycobacterium tuberculosis* in the lesion. Simple and fast complementary diagnosis by real-time polymerase chain reaction is a fundamental approach to establishing early and effective pharmacological and surgical treatment.

## Background

Tuberculosis is considered an emerging disease worldwide. The World Health Organization estimated that the incidence of tuberculosis increased in the last 10 years to more than 9.6 million cases of active tuberculosis, and that it caused 1.5 million patient deaths in 2014. Approximately 1 million children were infected by *Mycobacterium tuberculosis* and 140,000 children died as a result of tuberculosis. By the end of 2014, an additional 480,000 cases of multidrug-resistant tuberculosis were reported worldwide [1]. Tuberculosis is rarely confined to bone and develops frequently secondary to a systemic infection. However, oral presentation with bone involvement occurs in less than 3% of all reported cases; thus, it rarely arouses clinical suspicion at initial presentation. Mandibular osteomyelitis caused by *M. tuberculosis* infection is a rare medical condition. Because tuberculosis infection is rarely confined to bone tissue, such a manifestation may be considered as an extrapulmonary complication.

Adults are more frequently affected, although cases in children have also been described [2–5]. Tuberculous lesions of the oral cavity are quite rare; despite the high incidence of the systemic disease, they can be explained by *M. tuberculosis* inhibition by the salivary components [6]. The mechanism of propagation of tuberculosis infection to the mandibular bone can be by direct inoculation through dental extraction, lesions of the mucosa during teeth eruption, and spread from adjacent tissue or via a hematogenous route [7]. The clinical presentation and radiological imaging of mandibular tuberculosis are similar to those of chronic secondary osteomyelitis and regular dentoalveolar abscess. However, cervical lymphadenopathy produces discrete or diffuse masses, which are often not sensitive to palpation. These can be distinctive features in some patients [8]. This similarity to conventional cases of osteomyelitis emphasizes the importance of considering a differential diagnosis of tuberculous osteomyelitis of the jaw, especially if the patient has a suspicious medical record for tuberculosis [9, 10]. The clinical lesions of oral tuberculosis present as painful and irregular ulcers, especially in the posterior tongue area, pharynx, or palate, and they are frequently secondary to active pulmonary tuberculosis [11]. Bone involvement of the maxilla and mandible can produce tuberculous osteomyelitis by occult spread of the microorganism [12], and regional lymphadenopathy usually accompanies the oral lesions. For this reason, inclusion of scrofula in the differential diagnosis is obligatory [13]. Sometimes, bone invasion can lead to tuberculosis-related osteomyelitis. The incidence is higher in poor and densely populated areas, areas of low socioeconomic status, and in human immunodeficiency virus (HIV)-infected and immunocompromised individuals [11]. The differential diagnosis of oral tuberculosis includes other important diseases, such as squamous cell carcinoma; primary syphilis and various oral lesions, such as actinomycosis; fungal lung diseases, such as histoplasmosis, coccidioidomycosis, and blastomycosis; lymphoma; and submandibular sialadenitis [11]. It is important to remember that trauma is the leading cause of oral ulcers and should be included in the differential diagnosis, and all oral ulcers that raise suspicion of tuberculosis require biopsy to exclude cancer.

Radiographic features of acute osteomyelitis are usually not immediately apparent. First, the exudate progresses through the soft tissue component through the preexisting medullary spaces. Until the trabecular bone components have resorbed significantly, the magnitude of the destruction will be evident on radiographs as macular, mottled, or fuzzy in appearance with feathered edges and accompanied by unresorbed fragments of dead bone surrounded by large purulent areas. The chronic osteomyelitis area has a mottled appearance and is radio-opaque, which is designated as osteosclerosis that can be limited to the surrounding tooth root area. In other cases, it can induce osteosclerosis of larger areas or areas of edentulous bone [14]. *M. tuberculosis* induces a specific response in infected tissues that are characterized by local areas of macrophages surrounded by lymphocytes and fibroblasts, where the macrophages develop an abundant eosinophilic cytoplasm similar to the epithelial cells that are known to be like epithelioid cells. The fusion of macrophages gives them the appearance of giant Langerhans cells in which the nuclei are distributed in episomal form, characteristic of the histological diagnosis of bone tuberculosis [12].

## Case presentation

**Fig. 1:**
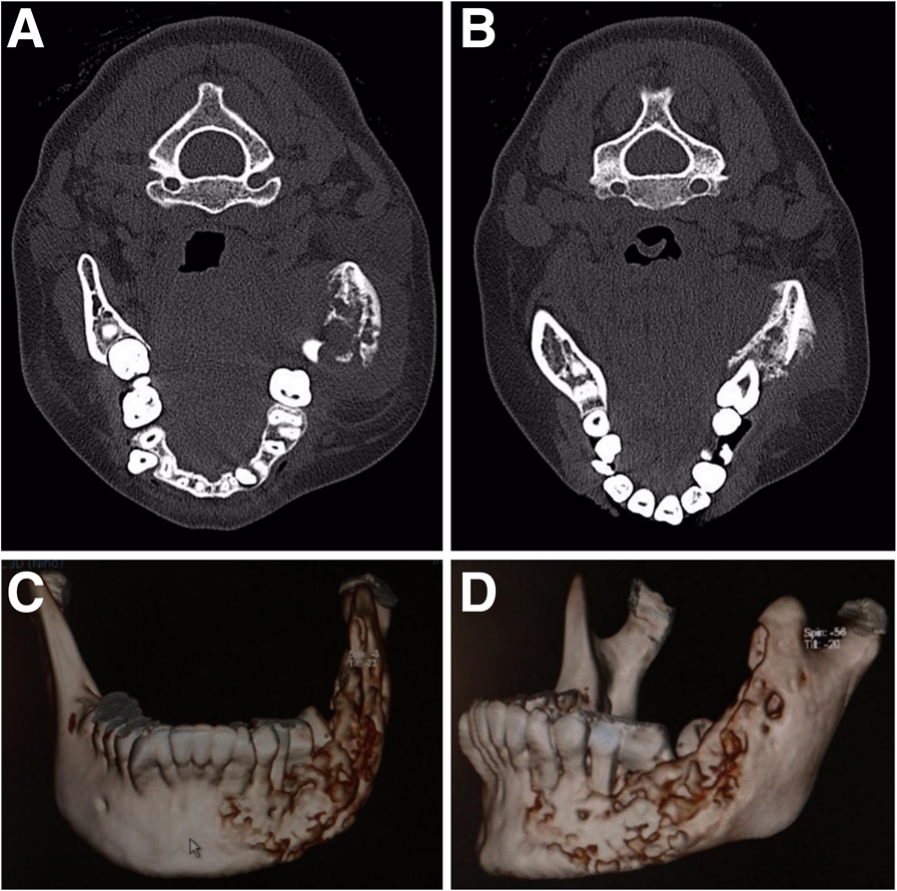
Clinical imaging of our patient with tuberculous osteomyelitis in the left mandible showing left-sided cheek swelling. (**a**) Frontal and (**b**) lateral views displaying mandible tuberculosis symptoms. (**c**) Frontal and (**d**) lateral views of three-dimensional reconstruction by computed tomography showing erosion and perforations of the cortical periosteal bone

**Fig. 2:**
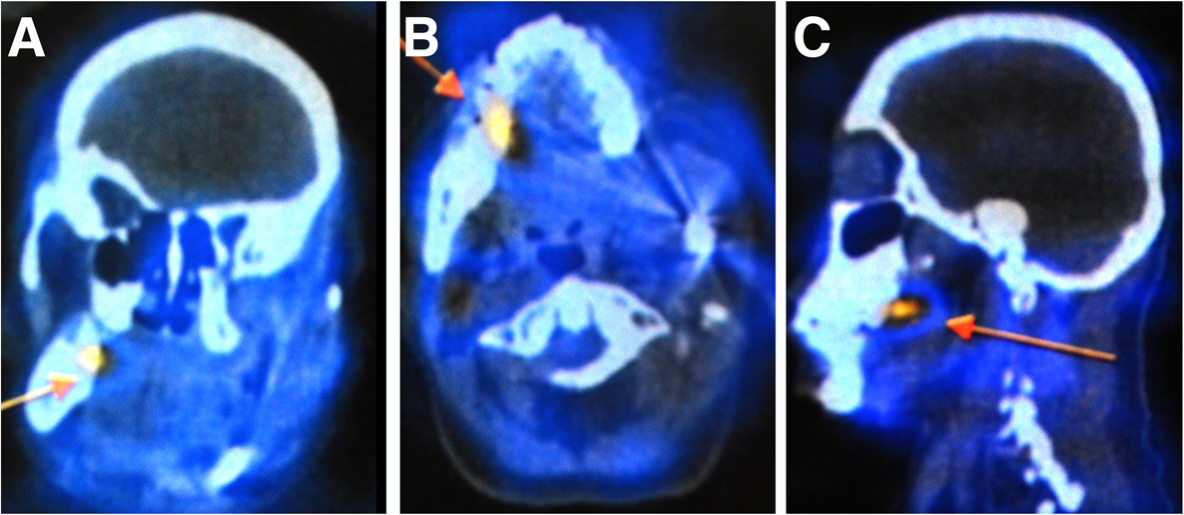
Technetium-99 m-ciprofloxacin accumulation in the mandible. Images show an abnormal concentrated area of radiolabeled antibiotic at the level of the left mandible, suggesting an infectious disease process. (**a**) Frontal view. (**b**) Oblique view. (**c**) Lateral view. The *arrows* indicate the site of 99mTc-Ciprofloxacine accumulation

A 15-year-old Mexican girl was referred to our hospital with a previous diagnosis of neurofibromatosis (NF), and reported pain and swelling in the region of the left mandibular body for the last 5 months. Her clinical history revealed antecedents of NF type 1, and her clinical examination disclosed pain in the mandibular region during palpation, a mass of soft consistency that seemed to involve bone, with a fistula and discharge of intraoral purulent material, tachycardia, and hyperthermia (39 °C). The left submental and submandibular regions had a 12-cm-diameter swelling and well-delineated, nonerythematous hyperthermia. An intraoral examination showed tooth mobility and few purulent exudate outputs. Laboratory studies provided the following relevant results: hemoglobin 12 g/dl, hematocrit 36.7%, and leukocytes 6.9 × 10^3^/μl. The differential white blood cell count showed neutrophils 4.2 × 10^3^/μl, lymphocytes 1.3 × 10^3^/μl, monocytes 1.1 × 10^3^/μl, eosinophils 0.3 × 10^3^/μl, basophils 0.09 × 10^3^/μl, and platelets 192 × 10^3^/μl. Computed tomography (CT) (Fig. 1a and b) and three-dimensional reconstruction by CT showed erosion and perforations of cortical periosteal bone formation of the left hemimandible (Fig. 1c and d). Single-photon emission computed tomography (SPECT) coupled with CT performed with technetium-99 m-ciprofloxacin (SPECT/CT ^99m^Tc-ciprofloxacin) revealed an abnormally concentrated area of radiolabeled antibiotic at the left mandible, right elbow, and soft tissues of the right hand, suggesting a disseminated infectious process (Fig. 2a–c). An intraoral secretion bacteriological culture isolated *Streptococcus parasanguinis* and high-level gentamicin- and vancomycin-resistant *Enterococcus faecium*. An antibiogram shows resistance to ampicillin, erythromycin, vancomycin, cefazolin, gentamicin, streptomycin, levofloxacin, oxacillin, and penicillin G, as well as sensitivity to daptomycin, doxycycline, linezolid, moxifloxacin, nitrofurantoin, quinupristin/dalfopristin, and rifampicin (RIF).

**Fig. 3:**
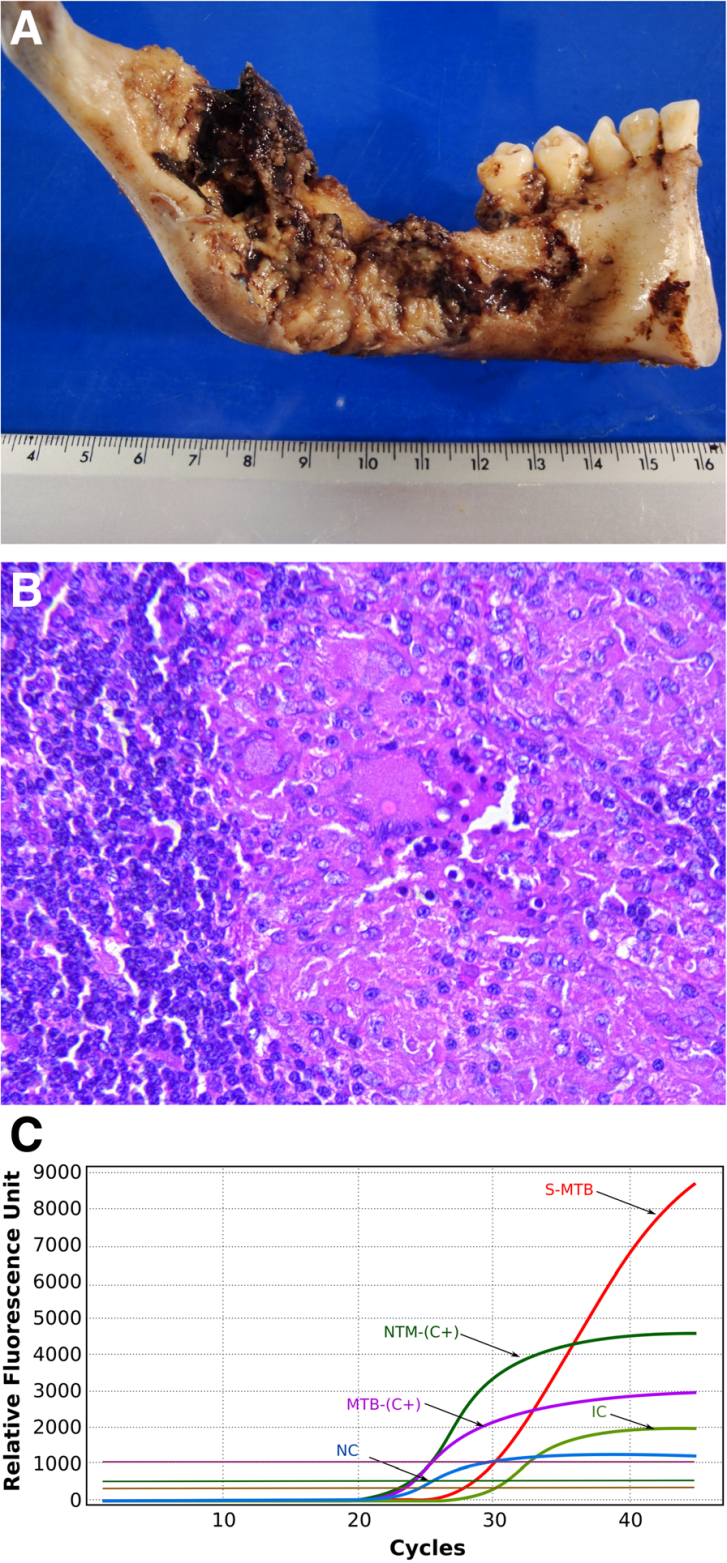
Macroscopic and histological characteristics of jaw and real-time polymerase chain reaction-based diagnosis. **a** Macroscopic characteristics of inner side of jaw. Large eroded areas accompanied by bleeding and bone decalcification can be seen. **b** Histopathological characteristics. Extensive chronic osteomyelitis process can be seen. **c** Molecular diagnosis of tuberculous osteomyelitis of the jaw. Both markers, susceptible *Mycobacterium tuberculosis* and *M. tuberculosis*-positive control, conformed to the *M. tuberculosis* diagnosis. *S-MTB* Susceptible *Mycobacterium tuberculosis* biopsy patient sample positive for *Mycobacterium tuberculosis*, *MTB-(C+) Mycobacterium tuberculosis*-positive control, *NTM-(C+)* Nontuberculous mycobacteria-positive control, *IC* Internal control, *NC* Negative control

The presumptive diagnosis was mandibular osteomyelitis. To corroborate the diagnosis, an incisional biopsy was done. The patient had received previous antibiotic administration with procaine benzylpenicillin (600,000 U intravenous therapy) combined with benzylpenicillin sodium (200,000 U intravenous therapy) and metronidazole (500 mg oral therapy) every 8 h for 7 days. Macroscopic and histopathological study revealed an extensive chronic osteomyelitic process (Fig. 3a and b) due to infection that did not cease with antibiotic administration. This raised the possibility of tuberculous osteomyelitis. To identify mycobacteria as a causative agent, molecular diagnostics for tuberculosis was performed for the high diagnostic value [15]. *M. tuberculosis* molecular identification and drug resistance to isoniazid (INH), RIF, fluoroquinolone (FQ), and aminoglycoside (AG) were done using Anyplex plus mycobacterium tuberculosis/nontuberculous mycobacteria/drug-resistant tuberculosis (MTB/NTM/DR-TB) real-time test (Seegene, Seoul, Korea) in accordance with the manufacturer’s specifications. The polymerase chain reaction (PCR) data confirmed the final molecular diagnosis of disseminated and chronic tuberculous osteomyelitis of the mandible caused by *M. tuberculosis* (Fig. 3c), and no isoniazid (INH) or rifampicin (RIF) resistance was found by the GenoType multidrug-resistant tuberculosis (MTBDR) test (Hain Lifescience, Nehren, Germany).

The patient received initial antituberculosis therapy with 300 mg INH, 600 mg RIF, 1.6 g pyrazinamide, and 1 g ethambutol (EMB) for 2 months, followed by maintenance treatment with 300 mg INH and 600 mg RIF. At the sixth month of tuberculosis therapy, the patient developed thrombosis of the inferior alveolar artery that produced extensive jawbone necrosis, which was considered irrecoverable. Thus, an elective hemimandibulectomy was performed, and the surgical defect was reconstructed by titanium plating with the condyle, and 1.2 g ETB was added to the antituberculosis maintenance therapy until 15 months of antifimic drug therapy was complete.

## Discussion

NF and tuberculosis rarely coincide in a patient; however, NF type 1 may be accompanied by a common variable immunodeficiency that favors infection by *M. tuberculosis* and its systemic dissemination [16]. In this paper, we report a case of a young woman with NF and disseminated tuberculosis affecting mainly the jaw that induced inferior alveolar artery thrombosis and irrecoverable mandibular necrosis requiring an elective hemimandibulectomy and completion of antifimic drug therapy.

Although bone tuberculosis, and especially the mandibular presentation [2–5], remains a rare disease it has great significance because of the significant facial deformity sequelae that occur. However, one of the dilemmas faced by the doctor is the definitive etiologic diagnosis (Table 1). In our patient’s case, different techniques used to support the clinical diagnosis included bacterial cultures, axial CT, and SPECT/CT ^99m^Tc-ciprofloxacin. All results obtained using these diagnostic techniques were indicative of infectious osteomyelitis. The incisional biopsy results were not conclusive for tuberculosis, because they showed only chronic osteomyelitis with an exacerbation process without evidence of granulomatosis. It is well known that all these techniques only have the power to suggest the type of pathology; however, the techniques required to make the diagnosis, one of which is mycobacteria cultivation, are not available in most hospitals. It is well known that this technique requires a long time to reveal the etiology of the disease. Furthermore, an average of 15 days to 5 weeks has typically been required to ultimately make the diagnosis on that basis. In our patient, the Löwenstein-Jensen culture medium was negative at 40 days of cultivation. However, with advances in technology using deoxyribonucleic acid (DNA) and real-time PCR techniques, microorganism identification now occurs in less time (8 h) after sampling. The resolving power that this technique offers is until a bacterial DNA molecule is present in the sample [17]. However, the sample type where *M. tuberculosis* was identified required a special procedure to amplify the bacterial DNA [15], because the paraffin-embedding process alters the DNA, and the calcium from the bone is capable of inhibiting the PCR enzymes, especially the DNA polymerase. For this reason, it was decided that the quantitative PCR (qPCR) technique should be used to increase the molecular diagnostic sensitivity [18]. For this purpose, a 90-bp segment was amplified, and its amplification was followed by hydrolysis with a TaqMan probe (Applied Biosystems, Foster City, CA, USA). According to the pathology results, the first site of infection manifestation was the mandible. This allowed us to hypothesize that the primary focus was mandibular tuberculosis, subsequently spread via the hematogenous mechanism to the left elbow and the soft tissues of the left hand; however, both foci were developed after mandibular infection without evidence of pulmonary tuberculosis. All tuberculosis infection foci also were corroborated by the qPCR technique.

**Table 1:** Comparative characteristics of reported tuberculous osteomyelitis of the jaw during infancy

Sex, age [reference]	Clinical findings	Mandibular Region	Histological diagnosis	Methods for tuberculosis diagnosis	Treatment (follow-up)
Male, 3 years old [2]	1 month Left submandibular mass Lymph enlargement	Angle	Granulomatous osteomyelitis consistent with tuberculosis	Primary. Ziehl-Neelsen staining and culturing not performed	Antitubercular therapy (6 months) Without recurrence
Male, 4 years old [3]	1 month Progressive swelling on cheek, right side Caries in the right second primary molar Lymph nodes	Body Angle	Granulomatous osteomyelitis	Secondary Granulomatous area Giant and epithelioid cells surrounded by lymphocytes and plasma cells Positive Ziehl-Neelsen staining	Antitubercular chemotherapy (6 months) Complete resolution
Female, 10 years old [4]	2 months Gradual swelling in left mandibular region Caries in the lower left second deciduous molar No evidence of lymphadenopathy	Body	Caseous granuloma	Primary Fine-needle aspiration cytology of swelling on left side of mandible	Antitubercular chemotherapy for 8 months Without recurrence
Female, 14 years old [5]	2 months Progressive swelling in the right parotid region Tooth extraction 8 months ago	Angle	Epithelioid cell granuloma with caseous necrosis Giant cell, Langerhans type Peripheral mantle of lymphocytes	Primary Open biopsy	Antitubercular therapy Complete resolution
Female, 18 years old [6]	8 months Edema and trismus (preauricular region) of left side of face Ulcerative lesion (retromolar region) Lymph node enlargement	Ramus Condyle	Caseous granuloma	Primary Fine-needle aspiration cytology	Not started
Female, 9 years old [7]	3 months Progressive mandibular edema Intermittent fever Lymph node	Angle Ramus	Multiple epithelioid cell granulomas Multinucleated giant cells Caseation necrosis areas Lymphocytic infiltrates	Primary Positive Ziehl-Neelsen staining for AFB	Antitubercular treatment (9 months) No recurrence
Female, 16 years old [8]	6 months No important antecedents No tooth extractions or any oral trauma	Ramus Condyle	Granulation tissue Necrotic bone Focal epithelioid cell granulomas Langerhans giant cells Chronic inflammatory cells	Primary Negative sputum for Ziehl-Neelsen staining Positive tuberculin skin test	Antitubercular treatment (9 months) No recurrence
Female, 16 years old [present report]	7 months Left mandibular inflammation Ulcerative lesion over the retromolar region (impacted 37) Lymph node	Body Ramus Condyle	Chronic osteomyelitic process	Primary Molecular diagnosis (real-time PCR)	Antitubercular treatment (9 months) Hemimandibulectomy Mandibular reconstruction No recurrence (15 months)

*AFB* Acid-fast bacilli, *PCR* Polymerase chain reaction

Our patient’s case highlights the clinical difficulty in establishing the etiology and simultaneously proposes the use of molecular diagnostics on the basis of clinical suspicion [2–5]. However, we are aware that not all hospitals have access to these advanced molecular diagnostic techniques. Establishment of an etiological diagnosis is the main goal of the clinic, with an aim to set up definitive treatment. Because a positive test for *M. tuberculosis* is necessary to initiate drug therapy in Mexico, as treatment is provided by the government free of charge for the patient, our patient underwent left hemimandibulectomy and placement of a mandibular reconstruction plate in the left jaw, accompanied by a three-drug antifimic scheme including INH (300 mg/d), RIF (600 mg/d), and ETB (1.2 g/d) 9 months after surgery, to which the patient responded satisfactorily. The hemimandibulectomy was considered as an optimal treatment because the patient had developed thrombosis of the inferior alveolar artery that had produced extensive jawbone necrosis, which was considered irrecoverable with the antituberculosis therapy. Currently, the patient is healthy and recovering, without indication of systemic tuberculosis. The clinical, surgical, and pharmacological results have allowed the patient to return to her daily activities, normal social life, and academic activities.

## Conclusions

The combination of NF and tuberculosis of the jaw is an extremely rare clinical condition with a high diagnostic difficulty grade. In our patient, the molecular diagnosis by qPCR played a key role in establishing the final diagnosis and surgical treatment as indicated, which allowed the patient to resume her normal activities and social relationships. Although primary tuberculous osteomyelitis of the jaw is a rare condition, it should be considered in the differential diagnosis of mandibular lesions in comparable clinical situations.

### Patient’s perspective

My parents and I are very happy and grateful for the care that I received from the medical staff of Pediatrics National Institute, Genomic Medicine Laboratory of the “October 1^st^” Regional Hospital, and in general, to all professionals who were involved to finally get my diagnosis, and mainly for solving my health problem.

## Abbreviations

^99m^Tc: Technetium-99 m
CT: Computed tomography
MTB: *Mycobacterium tuberculosis*
NTM: Nontuberculous mycobacteria
PCR: Polymerase chain reaction
qPCR: Quantitative polymerase chain reaction
SPECT: Single-photon emission computed tomography
TB: Tuberculosis
EMB: Ethambutol
INH: Isoniazid
RIF: Rifampicin
FQ: Fluoroquinolone
AG: Aminoglycoside
S-MTB: Susceptible *Mycobacterium tuberculosis*
MTB-(C+): *Mycobacterium tuberculosis*-positive control
NTM-(C+): Nontuberculous mycobacteria-positive control
IC: Internal control
NC: Negative control
NF: Neurofibromatosis
AFB: Acid-fast bacilli
